# Gender and Racial Distribution of Transthyretin Stabilizer Therapy in Patients With Transthyretin Cardiac Amyloidosis

**DOI:** 10.1016/j.jacadv.2026.103201

**Published:** 2026-10-21

**Authors:** Stephen J. Foley, Shruti Hegde

**Affiliations:** University of Massachusetts Chan Medical School, Worcester, Massachusetts, USA

**Keywords:** amyloid, tafamidis, transthyretin

**Figure:**
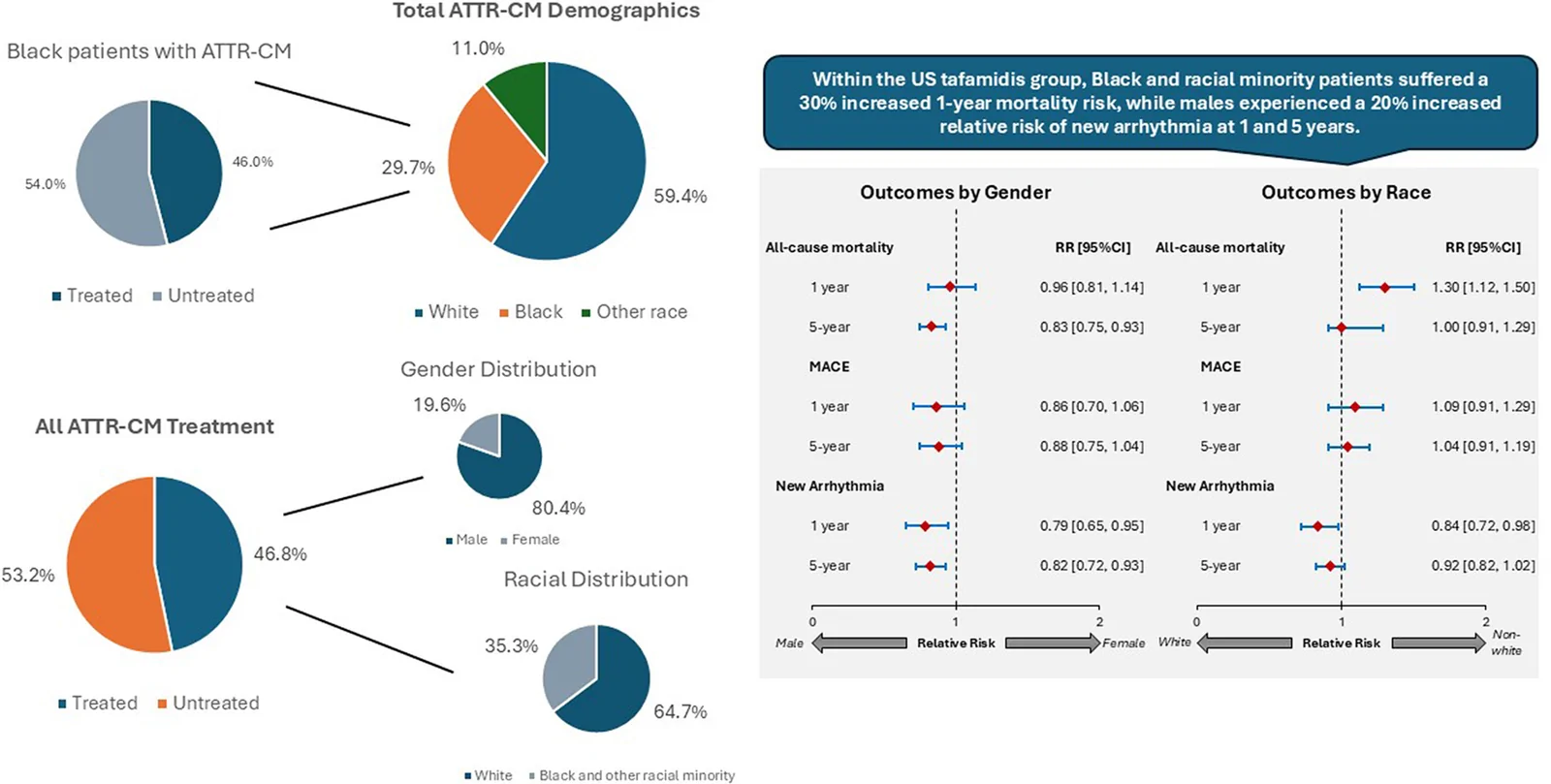

**What is the clinical question being addressed?**

How do current patterns of transthyretin stabilizer use across demographic groups relate to disparities in clinical outcomes among patients with ATTR-CM?

**What is the main finding?**

Racial minorities remain under-represented in ATTR-CM treatment groups and experience increased short-term mortality, despite no difference in cardiovascular events.

Transthyretin amyloid cardiomyopathy (ATTR-CM) is characterized by the misfolding, deposition, and accumulation of transthyretin amyloid fibrils within the myocardium. Progressive infiltration leads to significantly thickened and rigid ventricular walls, diastolic dysfunction, conduction abnormalities, and eventually heart failure.^1^ This increasingly under-recognized condition predominantly affects older adults, often diagnosed as restrictive cardiomyopathy in the eighth decade of life.^2^ Historically, median survival for these patients has been poor due to the limited management options besides supportive care and, in some circumstances, organ transplant.^1^^,^^2^ Recently, the therapeutic landscape has evolved with the innovation of transthyretin stabilizing agents, which have significantly extended patient survival and enhanced quality of life.^3^^,^^4^

Following the landmark ATTR-ACT (Transthyretin Amyloidosis Cardiomyopathy Clinical Trial) in 2018, tafamidis became established as first line therapy for ATTR-CM.^1^^,^^3^ This benzoxazole derivative binds to thyroxine-binding sites of transthyretin tetramers and inhibits monomer formation. Patients who received tafamidis experienced a marked reduction in all-cause mortality, slowed progression of left ventricular systolic and diastolic dysfunction, decreased cardiovascular-related hospitalizations, and significantly lower rate in functional decline.^1^^,^^3^ Later, the ATTRibute-CM study in 2024 unveiled acoramidis, a novel therapy with more potent and robust (>90%) ATTR stabilization efficacy, which similarly demonstrated marked improvement hierarchical composite of morbidity, mortality, and functional capacity.^4^ Here, our database-related study aims to provide updates in the current trends in gender and racial/ethnic distribution of ATTR-CM patients undergoing treatment with transthyretin stabilizers to promote improved representation and access to life-prolonging therapies.

This study’s data was collected from the TriNetX United States Collaborative Network, which provides access to electronic medical records (diagnoses, procedures, medications, laboratory values, and genomic information) from approximately 130 million patients from 73 health care organizations. This globally federated research database contains only aggregate, deidentified electronic medical record data per the deidentification standard defined in Section §164.514(a) of the HIPAA Privacy Rule. Therefore, this retrospective cohort study was exempt from approval by the University of Massachusetts Medical School institutional review board.

We queried the TriNetX database to generate cohorts of patients with documented history of ATTR-CM who were prescribed tafamidis between January 1, 2019 and March 1, 2026. Patients with cerebral amyloid angiopathy and light chain amyloidosis were excluded. Demographics and characteristics of cohort populations were collected. The primary objective of this cross-sectional analysis was to characterize the current gender and racial/ethnic distribution of patients currently prescribed tafamidis. We then stratified by race and gender to compare outcomes at 1 and 5 years, including all-cause mortality, major adverse cardiac event (MACE) (cardiac arrest, acute myocardial infarction, stroke, heart failure, or invasive cardiac procedure), and new arrhythmia (atrial fibrillation/flutter, ventricular fibrillation/flutter, any atrioventricular block, or bundle branch block). Outcomes analysis, including calculation of measures of association such as risk ratios (RRs) and HRs were calculated within TriNetX. Patients with prior history of these outcomes were excluded from analysis.

A total of 17,707 patients with ATTR-CM were identified (Figure 1). Of these, 8,279 (46.8%) had a documented tafamidis prescription and comprised the primary treatment cohort. An additional 504 patients were treated with acoramidis; however, they were excluded from the comparative outcomes analysis because of the smaller cohort size and the drug’s recent approval. The mean age of the tafamidis cohort at the time of analysis was 78.3 years. Overall, the cohort was predominantly male (80.4%) and White (64.7%). Compared to males, females had significantly higher rates of hypertension (72% vs 60%; *P* < 0.001), hyperlipidemia (65% vs 58%, *P* < 0.001), type 2 diabetes (32% vs 24%; *P* < 0.001), but lower rates of tobacco use (18% vs 23%; *P* < 0.001). Females also more often had diastolic heart failure at diagnosis (50% vs 38% in males; *P* < 0.001). In addition, compared to White patients, non-White patients were younger at diagnosis (mean age 76.3 vs 79.2 years; *P* < 0.001) with a higher representation of females (35% vs 13%; *P* < 0.001), and higher rates of hypertension (70% vs 61%; *P* < 0.001) and type 2 diabetes (38% vs 20%; *P* < 0.001). Non-White patients more often had systolic heart failure at diagnosis (44% vs 29% in White patients; *P* < 0.001). Lastly, approximately 54% of non-White patients were untreated during this study period.

Among tafamidis-treated patients, non-White patients demonstrated higher observed 1-year mortality (RR: 1.30; 95% CI: 1.12-1.50), whereas female patients demonstrated lower observed 5-year mortality (RR: 0.83; 95% CI: 0.75-0.93) (Figure 1). When censoring for patients lost to follow-up or lacking mortality data, racial differences in 1-year mortality were unchanged (HR: 1.35; 95% CI: 1.16-1.56), whereas gender differences in 5-year mortality lost significance (HR: 0.94; 95% CI: 0.83-1.06). Relative risk of MACE was similar between gender and racial cohorts at both time points. Non-White patients demonstrated a slight decrease in risk of new arrhythmia (RR: 0.84; 95% CI: 0.72-0.98), but female patients had a significantly lower risk of new arrhythmia at 1 year (RR: 0.83; 95% CI: 0.75-0.93) and 5 years (RR: 0.83; 95% CI: 0.75-0.93).

Despite these recent therapeutic advancements in transthyretin stabilizer therapies, notable gaps remain regarding the demographic distribution of clinical trial participants and treatment recipients. Both the ATTR-ACT and ATTRibute-CM studies found significant benefits of tafamidis and acoramidis prolonging life and improving quality of life, but the generalizability of their findings may be limited by cohorts that were predominantly male (approximately 90%) and White (approximately 80% to 88%).^1^^,^^3^^,^^4^ Compared to White patients, Black patients with ATTR-CM more often had the hereditary V122I variant (ATTRv-CM) but were diagnosed at later stages of disease with poorer functional status, leading to higher rates of left ventricular device placement, cardiac transplant, and death.^5^

Previous registry data demonstrated poorer outcomes among Black patients with ATTR-CM, although only 35% to 40% were receiving transthyretin stabilizer therapy.^5^ In our analysis restricted to tafamidis-treated patients, we observed no statistically significant racial difference in 5-year MACE. Nevertheless, over half of non-White patients in the broader ATTR-CM cohort had no documented prescription for a transthyretin stabilizer during the study period, highlighting a substantial gap in treatment uptake that warrants further investigation. Direct comparisons with prior studies should be interpreted cautiously because of differences in study populations, treatment exposure, baseline characteristics, and outcome definitions. Future studies should compare adjusted outcomes among treated and untreated patients within individual racial groups while accounting for disease severity, genotype, comorbidities, and treatment timing.

Finally, our analysis focused on transthyretin stabilizer therapy; however, 9.2% of tafamidis-treated patients also had a documented prescription for vutrisiran. Polyneuropathy was present in 38% and autonomic disorders in 5% of patients receiving concomitant therapy, consistent with clinical factors that may influence the use of a transthyretin silencer. Although concomitant vutrisiran use was relatively uncommon and therefore unlikely to have substantially influenced the overall cohort findings, prescribing patterns may vary across institutions and clinicians. The potential contribution of silencer therapy to observed outcomes cannot be fully excluded and should be evaluated in future studies using treatment-specific and time-dependent analyses.

In this retrospective analysis of a large U.S. electronic health record network, patients prescribed tafamidis were predominantly male and White. Non-White patients had higher observed unadjusted 1-year mortality, whereas composite cardiovascular event rates did not differ significantly by race. Because of baseline differences, incomplete genotype information, and the absence of risk adjustment, these findings should be considered hypothesis-generating and interpreted cautiously. Future studies should evaluate race-specific treatment uptake using appropriate denominators and adjusted time-to-event analyses to better identify potential barriers to equitable ATTR-CM care.

**Figure 1:**
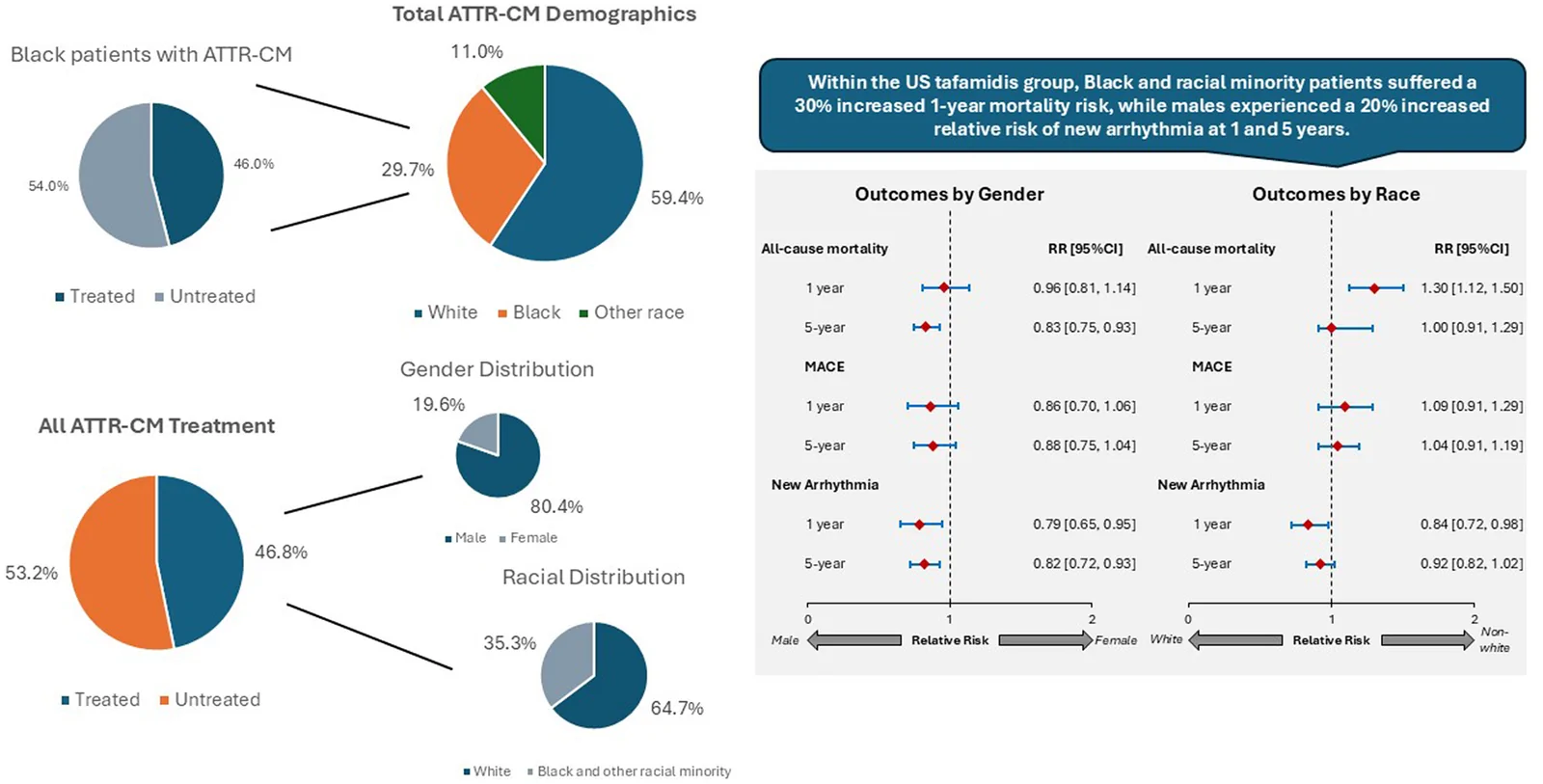
Gender and Racial Distribution of Tafamidis Use in ATTR-CM ATTR-CM = transthyretin amyloid cardiomyopathy; MACE = major adverse cardiac event; RR = risk ratio.

## Funding support and author disclosures

This study was funded by a research grant from Bridgebio. The authors have reported that they have no relationships relevant to the contents of this paper to disclose.
